# Analysis of Risk Factors for Major Complications of 1500 Transvenous Lead Extraction Procedures with Especial Attention to Tricuspid Valve Damage

**DOI:** 10.3390/ijerph18179100

**Published:** 2021-08-28

**Authors:** Łukasz Tułecki, Anna Polewczyk, Wojciech Jacheć, Dorota Nowosielecka, Konrad Tomków, Paweł Stefańczyk, Jarosław Kosior, Krzysztof Duda, Maciej Polewczyk, Andrzej Kutarski

**Affiliations:** 1 Department of Cardiac Surgery, The Pope John Paul II Province Hospital of Zamość, 22-400 Zamość, Poland; luke27@poczta.onet.pl (Ł.T.); konradtomkow@wp.pl (K.T.); 2 Department of Physiology, Pathophysiology and Clinical Immunology Collegium Medicum, The Jan Kochanowski University, 25-369 Kielce, Poland; 3 Department of Cardiac Surgery, Świętokrzyskie Cardiology Center, 25-369 Kielce, Poland; 4 2nd Department of Cardiology, Silesian Medical University, 41-808 Zabrze, Poland; wjachec@interia.pl; 5 Department of Cardiology, The Pope John Paul II Province Hospital of Zamość, 22-400 Zamość, Poland; dornowos@wp.pl (D.N.); paolost@interia.pl (P.S.); 6 Department of Cardiology, Masovian Specialist Hospital of Radom, 26-617 Radom, Poland; jaroslaw.kosior@icloud.com; 7 Department of Cardiac Surgery, Masovian Specialist Hospital of Radom, 26-617 Radom, Poland; kadeder@gmail.com; 8 Faculty of Medicine and Health Studies, Jan Kochanowski University, 25-369 Kielce, Poland; Maciek.polewczyk@gmail.com; 9 Department of Cardiology, Medical University of Lublin, 20-509 Lublin, Poland; a_kutarski@yahoo.com

**Keywords:** transvenous lead extraction, lead extraction-related major complications, cardiac/vascular wall tear, worsening tricuspid regurgitation

## Abstract

Background: Transvenous lead extraction (TLE) is a relatively safe procedure, but it may cause severe complications such as cardiac/vascular wall tear (CVWT) and tricuspid valve damage (TVD). Methods: The risk factors for CVWT and TVD were examined based on an analysis of data of 1500 extraction procedures performed in two high-volume centers. Results: The total number of major complications was 33 (2.2%) and included 22 (1.5%) CVWT and 12 (0.8%) TVD (with one case of combined complication). Patients with hemorrhagic complications were younger, more often women, less often presenting low left ventricular ejection fraction (LVEF) and those who received their first cardiac implantable electronic device (CIED) earlier than the control group. A typical patient with CVWT was a pacemaker carrier, having more leads (including abandoned leads and excessive loops) with long implant duration and a history of multiple CIED-related procedures. The risk factors for TVD were similar to those for CVWT, but the patients were older and received their CIED about nine years earlier. Any form of tissue scar and technical problems were much more common in the two groups of patients with major complications. Conclusions: The risk factors for CVWT and TVD are similar, and the most important ones are related to long lead dwell time and its consequences for the heart (various forms of fibrotic scarring). The occurrence of procedural complications does not affect long-term survival in patients undergoing lead extraction.

## 1. Introduction

Transvenous lead extraction (TLE) is now an integral part of the lead management strategy [1,2,3,4,5]. Fibrotic scarring around the leads [6] places the patient at risk of fatal complications such as venous or cardiac injury with severe bleeding [7,8,9,10,11] or worsening tricuspid regurgitation [12,13,14,15,16,17,18]. The problem of tricuspid valve damage was overlooked in several previous guideline revisions [1,2,3,4] and addressed only in the recent ones [4,5]. Up to date, several attempts have been made in search of the risk factors predictive of major complications [19,20,21,22]. Such knowledge is useful to plan the strategy of TLE including selection of the center, venue, first operator, organizational model (staging of safety precautions). Analysis of the well-and lesser-known factors facilitates the calculation of the real risk of major complications [23,24,25,26,27]. It also helps better prepare and provide preoperative information to the patient and family members. However, most of the available risk calculators had been invented, when worsening tricuspid regurgitation was not accepted officially as major complication of lead removal (before 2017). Recently, more and more investigators have paid attention to inadvertent tricuspid valve damage during TLE [12,13,14,15,16,17,18], and an analysis of risk factors that are specifically associated with this complication seems to be justified. Their identification, especially a history of pacing and previous lead management strategies may change our current routine and update the guidelines in the future.

The aim of this study was to determine circumstances of occurrence and risk factors (patient-dependent, pacing history-related, procedure-related) of cardiac/vascular wall tear (CVWT) and TV damage (TVD) considered as TLE major complication with focus on the utility of information obtained in monitoring by transesophageal echocardiography (TEE) during lead extraction.

## 2. Materials and Methods

This study was a post-hoc analysis of the clinical data of 1500 patients undergoing transvenous lead extraction at two high-volume centers between June 2015 and April 2021. We compared the clinical and procedure-related factors as well as echocardiographic findings in patients with major complications during lead extraction (with particular emphasis on cardiac/vascular wall damage and tricuspid valve damage) and in individuals without TLE-related complications.

The following clinical variables were taken into account: age, gender, NYHA class, renal failure and infectious indications for TLE. The procedure-related variables included type of the implanted system, the number and type of leads being extracted, as well as the risk for the occurrence of major complications measured as the SAFeTY TLE score [23]. The echocardiographic variables considered for the analysis included left ventricular ejection fraction (LVEF), the degree of tricuspid valve (TV) dysfunction before and after TLE, mean right ventricular systolic pressure (RVSP), the presence of fibrotic scarring, lead thickening, lead-to-lead binding, lead adherence to any heart structure and right ventricular wall perforation by the lead. The study subgroups were also compared with regard to the course of the procedure measuring TLE duration time (skin-to-skin and sheath-to-sheath duration), presence of lead-to-lead adhesions, occurrence of any technical problem during TLE, block at lead venous entry site, extracted lead fracture, Byrd dilator torsion/collapse, utility of specific tools such as Evolution, TightRail, lasso catheters/snares and need for temporary pacing during the procedure. Of the echocardiographic and hemodynamic monitoring parameters we compared pulling on the cardiac walls and other leads as well as a drop in blood pressure during TLE. This study also analyzed complete procedural and clinical success as well as short-and long-term survival (mortality at 1 month, 1 year, 3 years and >3 years after TLE).

### 2.1. Lead Extraction Procedure

Lead extraction procedure was defined according to the most recent guidelines on the management of lead-related complications (HRS 2017 and EHRA 2018) [2,3,4,5]. Indications for TLE and type of periprocedural complications were defined according to the 2017 HRS Expert Consensus Statement on Cardiovascular Implantable Electronic Device Lead Management and Extraction [4].

Most procedures were performed using nonpowered mechanical systems such as Byrd polypropylene dilator sheaths (Cook^®^ Medical, Leechburg, PA, USA), if only possible via the implant vein. If technical difficulties arose, alternative venous approaches or additional tools such as Evolution (Cook^®^ Medical, Leechburg, PA, USA), TightRail (Spectranetix, Sunnyvale, CA, USA), lassos, basket catheters were utilized. The excimer laser was not applied. 

All extraction procedures were performed following the same organizational model in accordance with the current guidelines. The operating team consisted of a very experienced extractor, cardiac surgeon, anesthesiologist and echocardiographist. The procedures were performed in a hybrid room or a cardiac surgery operating room, with a full range of equipment for an emergency rescue.

The SAFeTY TLE score was used to assess the risk for the occurrence of major complications related to TLE [23] using an online calculator, available at http://alamay2.linuxpl.info/kalkulator/ (accessed on 27 August 2021). The calculator is available on the website www.usuwanieelektro.pl. (accessed on 27 August 2021).

The following terms were used to assess the duration of the procedure: skin-to-skin time and sheath-to-sheath time. The skin-to-skin time is time in minutes from the cutting to the sewing of the skin. It includes not only dissection of the lead (s), but also lead re-implantation for non-infectious indications. The sheath-to-sheath time (in minutes) is total time for dissection and removal of all scheduled leads.

### 2.2. TEE Monitoring during TLE 

Transthoracic examinations (TTE) and transesophageal echocardiography monitoring were performed using Philips iE33 or GE Vivid S 70 machines equipped with X7-2t Live 3D or 6VT-D probes. All recordings were archived and, in accordance with the guidelines, included a preoperative examination, navigation during TLE, and postoperative evaluation of the effectiveness of the procedure with an assessment of possible complications. [28,29,30,31]. The projections and consecutive stages of echocardiographic monitoring were described in detail in previous publications [28,29,30,31]. The preoperative monitoring phase (TTE and TEE) included assessment of lead position, lead-to-lead binding and adhesions between the leads and the walls of the heart, the presence of additional masses on the leads, and evaluation of tricuspid valve function.

The intraoperative phase of TEE monitoring allowed visualization of direct pulling on the heart and the right ventricular cavity during lead removal. Often, a drop in blood pressure is observed, and monitoring makes it possible to clarify the cause of this phenomenon [28,29,30,31]. Additionally, it is possible to quickly assess damage to the heart wall with accumulation of excess fluid in the pericardial sac [29,30]. The post-procedural phase of TTE and TEE monitoring includes reassessment of cardiac/vascular wall injury and tricuspid valve function, detection of lead remnants and residual vegetations.

### 2.3. Statistical Analysis 

The Shapiro–Wilk test showed that most continuous variables were normally distributed. For uniformity, all continuous variables are presented as the mean ± standard deviation. The categorical variables are presented as number and percentage. The study population was divided into the following groups: A—patients with hemorrhagic complications due to cardiac/vascular wall tear; B—patients with tricuspid valve damage, C—patients from groups A and B, and D—patients without complications. The significance of differences between groups (A, B, C vs. D) was determined using the nonparametric Chi2 test with Yates’s correction or the unpaired “U” Mann–Whitney test, as appropriate. The Spearman r correlation was determined for pulling on vascular or cardiac structures during TLE and maximal drop in blood pressure. A *p*-value less than 0.05 was considered as statistically significant. 

Statistical analysis was performed with Statistica version 13.3 (TIBCO Software Inc., Palo Alto, CA, USA).

### 2.4. Approval of the Bioethics Committee

All patients gave their informed written consent to undergo TLE and to use anonymous data from their medical records, approved by the Bioethics Committee at the Regional Chamber of Physicians in Lublin no. 288/2018/KB/VII. The study was carried out in accordance with the ethical standards of the 1964 Declaration of Helsinki.

## 3. Results

The study population consisted of 1500 patients, mean age 68.11 years, 39.87% of females. The mean left ventricular ejection fraction (LVEF) was 49.26%, renal failure occurred in 25.00% of patients, the Charlson comorbidity index was 5.10. The indications for lead extraction included systemic infection (with pocket infection or not) in 15.33% of patients, local (pocket) infection in 6.00%, lead failure (replacement) in 57.67%, change of pacing mode/upgrading/downgrading in 7.33%, other in 12.87% of patients. Overall, 67.07% of patients had a pacemaker, 23.93% cardioverter-defibrillator (ICD), and 8.87% resynchronization device (CRT-D). The dwell time of the oldest lead per patient before TLE was 112.1 (months), the cumulative lead dwell time before TLE was 17.01 (years).

Patients with hemorrhagic complications (cardiac/vascular wall tear) were significantly younger and received their first cardiac implantable electronic device (CIED) 15 years earlier than in the control group. There were twice as many women as men, and significantly fewer patients with low LVEF and in NYHA class III or IV. The Charlson comorbidity index was much lower as compared to the control group. The indications for TLE were comparable to the remaining groups of patients (Table 1). 

Patients with TV damage (TVD group) were older compared to the other subgroups but received their first CIED nine years earlier. There were fewer women, and similarly to CVWD group, there were fewer patients with low LVEF and in NYHA class III or IV. The Charlson comorbidity index was slightly lower as compared to the control group. The indications for TLE were comparable to CVWD and control groups. TVD patients frequently had an abandoned lead, more CIED-related procedures and more often longer implant duration similar to CVWT patients (Table 1).

The number of extracted leads per patient (*p* = 0.056), the need to extract three or more leads, extraction of leads with redundant loops, extraction of abandoned lead (s) and extraction of atrial leads were regarded as intraprocedural risk factors for CVWT and TVD. There was one exception, however. Atrial lead extraction was strongly associated with CVWT but not with TV damage, and extraction of abandoned leads was more likely to be related to CVWT (Table 2).

Implant duration was the strongest predictor of both CVWT and TVD. An interesting finding was the value of the SAFeTY TLE score estimating the risk of procedure. The calculator had been created before 2017 when TVD was not considered as major complication; it works excellently and the calculated (automatically) risk of CVWT and TVD was 5.2-fold and 3.4-fold higher than in the control group.

Passive fixation leads were also predictors of CVWT and TVD (RAA tear was the most frequent finding) (Table 2).

Preoperative TTE and TEE demonstrated that the state of the tricuspid valve was similar in groups with major complications of TLE. These groups were characterized by higher LVEF and lower RVSP. TEE before TLE provided much more valuable information. Oscillating scar tissue on the leads, lead thickening, lead-to-lead binding, lead adhering to any heart structure, lead adhering to the tricuspid valve, to the walls of the superior vena cava (SVC), right atrium (RA) and right ventricle (RV), the presence of any form of scar tissue were much more often detected in the two groups with major complications. Additionally, all forms of scar tissue were more frequent in patients with postprocedural TVD. However, there was one exception: a small percentage of leads adhering to the RA wall in patients with postprocedural TVD (Table 3).

Our analysis of the effectiveness and TLE-related complications demonstrated that procedure duration (skin-to-skin time, sheath-to-sheath time) and mean extraction time per lead were much longer in patients with the two types of major complications. The occurrence of any technical problem during TLE, lead-to-lead binding (intraoperative diagnosis), fracture of the extracted lead, three or more technical problems, the need to use Evolution or TightRail or lasso catheters/snares were dramatically more frequent in groups with CVWT or TVD. It seems to be related to lead implant duration, proliferation of tissue scar around the lead and necessity to use slightly more aggressive tools. Byrd dilator torsion/collapse is more frequent if ventricular leads are extracted, which is easy to explain by the anatomy (bend) and extracted lead route (Table 4).

TEE and blood pressure monitoring during TLE showed that pulling on the right atrial appendage (RAA), TV and RV wall as well as pulling on the other lead were more common in patients with CVWT and TVD. A transient drop in blood pressure during TLE is usually caused by pulling on the RV wall, rarely on the SVC with a significant reduction of its diameter or by any reflex action (Spearman rank correlation coefficient r = 0.320; *p* < 0.001). This was confirmed by the drop in blood pressure in TVD group vs. control group. However, a decrease in blood pressure can be a warming sign of bleeding into the pericardial sac, right pleura or mediastinum. The drop in blood pressure was significantly higher in patients with CVWT because of blood loss (Table 4).

Assessment of TV function during and after TLE revealed that the worsening of tricuspid valve regurgitation (TVR) by one degree was similar in all study subgroups. TVD after TLE was considered as major complication if TR deteriorated by at least two degrees to grade 4.

Irrespective of the organizational model of TLE procedures and despite the occurrence of severe major complications, there were no procedure-related deaths. Effective surgical management of CVWT resulted in the rates of clinical and procedural success comparable to those in the control group. 

An analysis of short-, mid-and long-term mortality (for any reason) after TLE demonstrated that there were no deaths within 30 days. Mid-and long-term mortality in patients with major complications was similar to that in the control group (Table 4).

**Figure 1:**
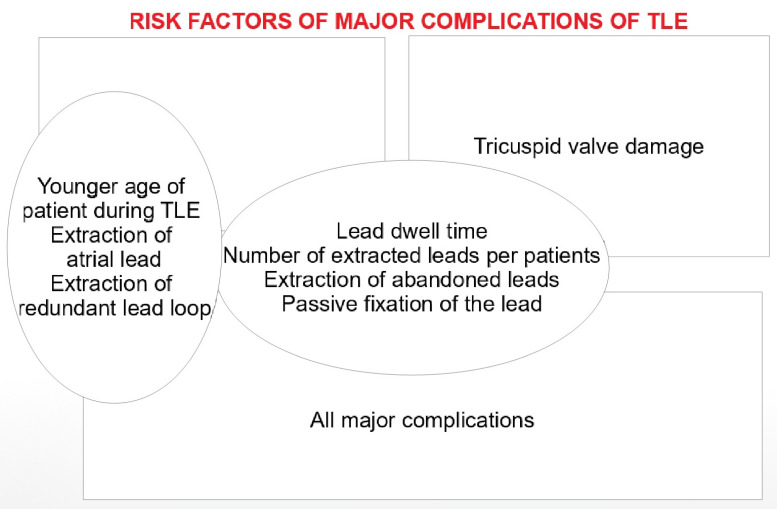
Graphical presentation of the main results

A summary of the most important risk factors for TLE complications is presented schematically in Figure 1.

## 4. Discussion

Transvenous lead extraction is an integral part of the management of CIED-related problems [1,2,3,4,5]. Cardiac and venous injuries during lead extraction are complications with potentially serious consequences. So far, there has been no comprehensive analysis of TLE complications that would include TLE-related TV damage apart from injuries to the SVC/other vessels. Not much is known about risk factors for TLE-related TV damage [12,13,14,15,16,17,18].

This study showed that patients with hemorrhagic complications were significantly younger and received their first CIED earlier than in the control group. There were twice as many women as men, among them significantly fewer patients with low LVEF and class NYHA class III or IV, and they were more likely to have procedural risk factors (abandoned leads, excessive loops of the leads, more previous CIED-related procedures). The younger age of the patient during TLE is one of the risk factors, especially for CVWT, because in young people, more intense proliferation of connective tissue is observed, with more adhesion of the lead to the walls of the heart and vessels. This factor, as well as lead dwell time and female gender, was included in the previously constructed risk scale of major complications SAFeTY-TLE [23].

Patients with worsening TVR were older but received their CIED nine years earlier than the control group. In many ways, patients with TVD are somewhere between those with CVWT and the control group. The number of leads extracted per patient, need to extract three or more leads, extraction of leads with redundant loops, extraction of abandoned lead(s) and extraction of atrial leads were intraprocedural risk factors for CVWT and TVD; however, extended implant duration was the strongest predictor of both CVWT and TVD. Extraction of RAA leads, bipolar (BP) or unipolar (UP), and passive fixation leads indicated the risk of CVWT (the most frequent finding was RAA tear). Right ventricular lead tips placed in a different position than the apex, passive fixation and UP leads were the predictors TVD.

In the two groups with major complications the state of the tricuspid valve at baseline was comparable, LVEF was higher and right ventricular systolic pressure was lower. Moreover, patients with CVWT or TVD were significantly more likely to have any form of tissue scar (oscillating tissue scar on the lead, lead thickening, lead-to-lead binding and lead adherence to any heart structure). Procedure duration was much longer in patients with the two types of major complications. The occurrence of any technical problem during TLE, lead-to-lead binding (intraoperative diagnosis), fracture of extracted leads, three or more technical problems, need to use Evolution (old and new) or TightRail or lasso catheters/snares were dramatically more frequent in patients with CVWT or TVD. A frequent technical problem during complicated TLEs was lead breakage. This is probably related to similar risk factors, as shown in the literature [32].

Pulling on the RAA, TV and RV wall as well as other lead was more common in patients with CVWT and TVD. A transient drop in blood pressure during TLE is usually caused by pulling on the RV wall, rarely on the SVC with a significant reduction of its diameter or by any reflex action. The BP drop was significantly higher in patients with CVWD because of blood loss.

According to recent reports, the use of a laser is associated with high efficiency also in the removal of leads with a long dwell time, although the rate of major complications remains relatively high (3.3%) [33]. If excimer laser energy is not applied, major complications other than tear of the SVC and anonymous vein seem to be more common [21,22,23,24,25,26,27]. The available guidelines and medical literature focus on cardiac/vascular wall tear but not on worsening TR after TLE [1,2,3,4,5,7,8,9,10,11]. On the other hand, several reports (experience with 100–200 TLE procedures) have described a wide spectrum of TLE-related TVD (Givon—15% [14], Park—11.5% [12], Franceschi—9.1% [18], Rodriguez—6% [17], Coffey—5.6% [16], Pecha—1.9% [13], Regoli—1.2% [15]), but there has been little discussion about risk factors for TLE-related TVD. In the 2018 EHRA expert consensus statement on lead extraction—recommendations on definitions, endpoints, research trial design, and data collection requirements for clinical scientific studies and registries: endorsed by APHRS/HRS/LAHRS [5]—we find much lower percentages: flail tricuspid valve leaflet requiring intervention: 0.03% (being major complication) and worsening tricuspid valve function: 0.02–0.59 % (being minor complication).

There are two large studies of the occurrence and management of cardiac/vascular wall damage (CVWD) during lead extraction using mainly laser technique [7,9]. Brunner et al. reported a 0.8% incidence rate of complications requiring rescue intervention (mean implant duration time 4.9 years). SVC laceration was most frequent (80%), whereas RA and RV wall damage was rare [7]. Bashir et al. reported CVWD in 3% of TLE patients, but mean implant duration time was much longer than in the previous study (10.8 years). Overall, 84.8% of devastating injuries were cardiac tamponade [9].

Damage to the tricuspid valve during extraction is estimated to range from 3.5% to 15%, and even to 19% [4,5,12,13,14,15,16,17,18]. In this study, we noted worsening TR by 1 degree (7.29%), by 2 degrees (2.50%), by 3 degrees (0.61%) and severe TVD fulfilling the criteria of TV repair (0.82%), which is less than previously reported [4,5,12,13,14,15,16,17,18]. The need for surgical intervention in such cases is rare [12,13,14,15,16,17,18,34].

This study and literature review [12,13,14,15,16,17,18,34] indicate that one of most important safety challenges during lead removal is still the unsolved problem of TLE-related TV damage which is caused by fibrous adhesion of the lead to the TV leaflet. Excessive pulling on the lead may cause leaflet disruption, and wrapping of the leaflet around the dilating sheath during rotational lead extraction will do the same. Excellent teamwork combined with TEE monitoring may help warn the extractor about potentially harmful situations leading to TV damage [28,29,30,31]. The lead to be removed can be fused to the chordae tendinae or even to the head of the papillary muscle and damages to these structures may go unnoticed. According to recent report, monitoring of TLE by intracardiac echocardiography may even more precisely visualize the growth of he leads to the walls of the heart, including the tricuspid valve [35].

## 5. Conclusions

The risk factors for cardiac/vascular wall tear and tricuspid valve damage during TLE are similar and include extended implant duration and other procedural and system-dependent factors: number of extracted leads, extraction of leads with redundant loops, extraction of abandoned lead (s), extraction of atrial leads. The immediate cause of major complications is increased proliferation of the connective tissue resulting from the long presence of the leads in the heart and making them grow into the heart structures. Nevertheless, TVD patients are similarly old as the control group—proliferation of tissue scar surrounding the lead is similar to that observed in much younger patients with CVWD.

Both TVD and CVWT occur more frequently during extraction of pacemaker passive (and unipolar) fixation leads. ICD lead extraction does not generate higher risk of TVD or CVWT. The occurrence of TLE complications does not affect the long-term survival of patients.

## 6. Study Limitations

The database of the study group was integrated prospectively, but analysis was performed retrospectively. The main limitation is the lack of echocardiographic follow-up with late reassessment of TVD.

## Figures and Tables

**Table 1:** Patient/system/history of pacing.

	Hemorrhagic Complication (Cardiac/Vascular Wall Tear)	Tricuspid Valve Damage	All Major Complications (Mixed Damages 1 Case)	Control Group (No Major Complications)
Groups of patients	A *N* = 22 (1.5%) Mean ± SD *n* (%)	B *N* = 12 (0.8%) Mean ± SD *n* (%)	C *N* = 33 (2.2%) Mean ± S *n* (%)	D *N* = 1467 Mean ± SD *n* (%)
**Patients**				
Patient age during TLE [years]	63.14 ± 13.91 *p* = 0.009	68.75 ± 21.98 *p* = 0.116	65.82 ± 16.84 *p* = 0.005	68.16 ± 13.96
Patient age at first implantation [years]	45.32 ± 16.98 *p* < 0.001	50.58 ± 26.16 *p* = 0.007	47.97 ± 20.23 *p* < 0.001	59.06 ± 15.58
Sex (% of female patients)	17 (77.30) *p* = 0.004	5 (41.70) *p* = 0.979	21 (63.60) *p* = 0.005	555 (37.80)
NYHA class III & IV (%)	1 (4.50) *p* = 0.192	0 (0.00) *p* = 0.227	1 (3.00) *p* = 0.052	256 (17.50)
LVEF < 40%	1 (4.50) *p* = 0.003	2 (16.70) *p* < 0.001	3 (9.10) *p* < 0.001	555 (37.80)
Renal failure (any)	3 (13.60) *p* = 0.316	1 (8.30) *p* = 0.127	4 (12.10) *p* = 0.127	371 (25.30)
Charlson comorbidity index [points]	2.55 ± 2.41 *p* < 0.001	3.67 ± 3.53 *p* = 0.013	3.03 ± 2.85 *p* < 0.001	5.14 ± 3.76
**TLE Indications**				
CIED-related infection (any)	4 (18.20) *p* = 0.917	2 (16.70) *p* = 0.964	6 (18.20) *p* = 0.817	314 (21.40)
Non-infectious indications	18 (81.80) *p* = 0.917	10 (83.30) *p* = 0.964	27 (81.80) *p* = 0.817	1153 (78.60)
**System**				
Pacemaker-with RA lead	18 (81.80) *p* = 0.028	8 (66.70) *p* = 0.621	26 (78.80) *p* = 0.012	812 (55.40)
Pacemaker-without RA lead and only abandoned PM lead	2 (9.10) *p* = 0.974	3 (25.00) *p* = 0.294	4 (12.10) *p* = 0.663	164 (11.20)
ICD-with RA lead	0 (0.00) *p* = 0.170	0 (0.00) *p* = 0.424	1 (3.00) *p* = 0.210	170 (11.60)
ICD-without RA lead and only HV lead	1 (4.50) *p* = 0.409	1 (8.30) *p* = 0.982	1 (3.00) *p* = 0.379	187 (12.70)
ICD-CRT-D pacing system	1 (4.50) *p* = 0.726	0 (0.00) *p* = 0.562	1 (3.00) *p* = 0.377	132 (9.90)
Number of leads in the heart before TLE	2.14 ± 0.94 *p* = 0.690	2.08 ± 0.67 *p* = 0.684	2.15 ± 0.83 *p* = 0.365	1.92 ± 0.69
Abandoned leads before TLE	5 (22.70) *p* = 0.019	4 (33.30) *p* = 0.004	9 (27.30) *p* < 0.001	106 (7.20)
Large lead loop on X-rays before TLE	3 (13.60) *p* = 0.015	1 (8.30) *p* = 0.754	4 (12.10) *p* < 0.001	39 (2.70)
Small lead loop on X-rays before TLE	5 (22.70) *p* = 0.250	1 (8.30) *p* = 0.978	6 (18.20) *p* = 0.452	180 (12.30)
Number of procedures before lead extraction	3.00 ± 2.00 *p* < 0.001	2.83 ± 1.34 *p* = 0.003	2.90 ± 1.77 *p* < 0.001	1.79 ± 0.91
Dwell time of the oldest lead per patient before TLE [months]	214.9 ± 91.86 *p* < 0.001	217.9 ± 106.2 *p* < 0.001	215.0 ± 96.87 *p* < 0.001	109.8 ± 76.15
Mean implant duration (per patient) before TLE [months]	201.25 ± 81.14 *p* < 0.001	178.0 ± 62.89 *p* < 0.001	191.4 ± 75.59 *p* < 0.001	103.3 ± 68.79
Global implant duration (sum of lead dwell times) [years]	36.96 ± 23.12 *p* < 0.001	30.56 ± 13.85 *p* < 0.001	35.12 ± 20.50 *p* < 0.001	16.60 ± 13.29

Abbreviations: CIED—cardiac implantable electronic device, CRT—cardiac resynchronization therapy, ICD—implantable cardioverter-defibrillator, LVEF—left ventricular ejection fraction, NYHA—New York Heart Association class, PM—pacemaker, RA—right atrium, TLE—transvenous lead extraction.

**Table 2:** Patient/system/history of pacing.

	Hemorrhagic Complication (Cardiac/Vascular Wall Tear)	Tricuspid Valve Damage	All Major Complications (Mixed Damages 1 Case)	Control Group (No Major Complications)
	A *N* = 22 Mean ± SD *n* (%)	B *N* = 12 Mean ± SD *n* (%)	C *N* = 33 Mean ± SD *n* (%)	D *N* = 1467 Mean ± SD *n* (%)
**TLE Procedure Potential Risk Factors of Major TLE Complications and Procedure Complicity**				
Number of leads extracted per patient	2.30 ± 1.58 *p* = 0.079	2.39 ± 1.81 *p* = 0.189	2.21 ± 1.34 *p* = 0.008	1.63 ± 0.71
Three or more leads extracted	5 (22.79) *p* = 0.091	2 (16.70) *p* = 0.739	7 (21.20) *p* = 0.056	141 (9.60)
Extraction of leads with redundant loop (large)	3 (13.60) *p* = 0.083	1 (8.30) *p* = 0.696	4 (12.10) *p* = 0.004	35 (2.40)
Extraction of abandoned lead(s) (any)	4 (18.20) *p* = 0.094	4 (33.30) *p* = 0.002	8 (24.20) *p* < 0.001	99 (6.70)
HV therapy (ICD) lead extracted	2 (9.10) *p* = 0.043	1 (8.30) *p* = 0.158	3 (9.10) *p* = 0.010	462 (31.50)
Atrial lead extracted (any)	19 (86.40) *p* = 0.018	6 (50.00) *p* = 0.080	25 (75.80) *p* = 0.044	867 (59.10)
CS (LV pacing) lead extracted	1 (4.50) *p* = 0.964	0 (0.00) *p* = 0.737	1 (3.00) *p* = 0.640	97 (6.60)
Dwell time of the oldest lead extracted	214.9 (91.86) *p* < 0.001	217.9 ± 106.2 *p* = 0.001	215.0 ± 96.87 *p* = 0 < 001	108.8 ± 75.74
Average (per patient) dwell time of lead extracted	201.3 (81.14) *p* < 0.001	176.9 ± 63.75 *p* = 0.001	191.0 ± 75.91 *p* < 0.001	103.8 ± 69.47
Cumulative dwell time of lead extracted (in years)	36.34 (23.81) *p* < 0.001	28.72 ± 14.88 *p* = 0.001	34.05 ± 21.37 *p* < 0.001	14.96 ± 13.23
SAFeTY TLE calculator of risk of MC of TLE—[number of points]	13.03 (4.73) *p* < 0.001	11.42 ± 4.60 *p* = 0.001	12.31 ± 4.69 *p* < 0.001	6.11 ± 4.32
Risk of MC calculated by SAFeTY TLE calculator (%)	9.40 (12.70) *p* < 0.001	6.17 ± 6.06 *p* < 0.001	8.06 ± 10.89 *p* < 0.001	1.79 ± 2.58
**Analysis of Extracted Leads: Lead Model, Tip Location and Mechanism of Tip Fixation.**				
**Tip Location**				
RAA	22 (47.73) *p* = 0.139	7 (30.43) *p* = 0.355	29 (42.64) *p* = 0.355	901 (37.04)
BB	1 (2.27) *p* = 0.693	0 (0.00) *p* = 0.911	1 (1.47) *p* = 0.911	15 (0.62)
CS	1 (2.27) *p* = 0.967	0 (0.00) *p* = 0.811	1 (1.47) *p* = 0.811	25 (1.03)
CSO	1 (2.27) *p* = 0.772	0 (.00) *p* = 0.783	1 (1.47) *p* = 0.783	44 (1.82)
RVA	17 (38.64) *p* = 0.505	10 (43.48) *p* = 480	27 (39.71) *p* = 0.483	1069 (43.69)
Outside RVA	2 (4.55) *p* = 0.231	6 (26.09) *p* = 0.985	8 (11.76) *p* = 0.985	274 (11.31)
LV vein	1 (2.27 *p* = 0.726	0 (0.00) *p* = 0.390	1 (1.47) *p* = 0.390	108 (4.46)
**Lead Type**				
BP pacemaker leads	39 (86.67) *p* = 0.106	18 (78.26) *p* = 0.146	57 (83.82) *p* = 0.146	1828 (75.04)
VDD pacemaker leads	0 (0.00) *p* = 0.952	0 (0.00) *p* = 0.730	0 (0.00) *p* = 0.730	30 (1.19)
UP pacemaker leads	4 (8.89) *p* = 0.256	4 (17.39) *p* = 0.007	8 (11.76) *p* = 0.007	104 (4.19)
ICD leads single coil	2 (4.44) *p* = 0.231	0 (0.00) *p* = 0.053	2 (2.94) *p* = 0.053	274 (11.25)
ICD leads dual coil	0 (0.00) *p* = 0.084	1 (4.35) *p* = 0.077	1 (1.47) *p* = 0.077	200 (8.21)
All	45 (100)	23 (100.0)	68 (100.0)	2436 (100.0)
**Tip Fixation Mode**				
Active fixation lead	9 (20.00) *p* < 0.001	11 (47.83) *p* < 0.001	20 (29.41) *p* < 0.001	1408 (57.73)
Passive fixation lead	36 (80.00) *p* ≤ 0.001	12 (52.17) *p* < 0.001	48 (70.59) *p* < 0.001	1028 (42.18)

Abbreviations: BB—Bachman Bundle, BP—bipolar, CS—coronary sinus, CSO—coronary sinus ostium, ICD—implantable cardioverter-defibrillator, LV vein—cardiac vein located on LV wall utilized for LV pacing PM—pacemaker, RA—right atrium, RAA-right atrial appendage, RVA—RV apex, UP—unipolar, VDD—single-lead atrial triggered ventricular pacing, TLE—transvenous lead extraction.

**Table 3:** TTE and TEE before TLE.

	Hemorrhagic Complication (Cardiac/Vascular Wall Tear)	Tricuspid Valve Damage	All Major Complications (Mixed Damages 1 Case)	Control Group (No Major Complications)
Groups of patients	A *N* = 22 Mean ± SD *n* (%)	B *N* = 12 Mean ± SD *n* (%)	C *N* = 33 Mean ± SD *n* (%)	D *N* = 1467 Mean ± SD *n* (%)
**TTE before TLE**				
LVEF average [%]	59.43 ± 10.85 *p* = 0.002	56.00 ± 11.78 *p* = 0.049	58.06 ± 11.29 *p* < 0.001	49.07 ± 15.96
TVR-mild (0,1)	15 (68.20) *p* = 0.214	8 (66.70) *p* = 0.214	22 (66.70) *p* = 0.153	771 (52.60)
TVR-intermediate/mid (2,3)	6 (27.30) *p* = 0.417	4 (33.30) *p* = 0.469	10 (30.30) 0.469	558 (38.00)
TVR-severe (4)	0 (0.00) *p* = 0.382	0 (0.00) *p* = 0.215	0 (0.00) *p* = 0.215	104 (7.10)
Lack of examination	1 (4.50) *p* = 0.610	0 (0.00) *p* = 0.997	1 (3.00) *p* = 0.997	67 (4.60)
RVSP [mm Hg]	27.24 ± 8.57 *p* = 0.075	26.08 (7.12) *p* = 0.010	27.06 ± 7.98 *p* = 0.010	32.07 (11.82)
**TEE Findings before TLE**				
Oscillating tissue scar on the lead	7 (38.80) *p* = 0.080	3 (25.00) *p* = 0.044	10 (30.30) *p* < 0.044	231 (15.70)
Lead thickening (encapsulation)	14(63.60) *p* < 0.001	9 (75.00) *p* < 0.001	22 (66.70) *p* < 0.001	398 (27.10)
Lead-to-lead binding	10 (45.50) *p* < 0.001	5 (41.70) *p* < 0.001	15 (45.50) *p* < 0.001	208 (14.20)
Lead adhering to any heart structure	11 (50.00) *p* < 0.001	10 (83.30) *p* < 0.001	20 (60.60) *p* < 0.001	242 (16.50)
Lead adhering to tricuspid valve	5 (22.70) *p* = 0.031	6 (50.00) *p* < 0.001	11 (33.30) *p* < 0.001	115 (7.80)
Lead adhering to superior vena cava	5 (22.70) *p* = 0.004	4 (33.30) *p* < 0.001	9 (27.30 *p* < 0.001)	83 (5.70)
Lead adhering to RA wall	9 (40.90) *p* < 0.001	1 (8.30) *p* < 0.001	10 (30.30) *p* < 0.001	92 (6.30)
Lead adhering to RV wall	7 (31.80) *p* = 0.002	8 (66.70) *p* < 0.001	14 (42.40) *p* < 0.001	140 (9.50)
Tissue scar occurrence (any form) (possible multiple options)	3.272 ± 1.725 *p* < 0.001	3.917 ± 1.647 *p* < 0.001	3.515 ± 1.587 *p* < 0.001	1.188 ± 1.225
Occurrence of any form of tissue scar	14 (63.60) *p* = 0.026	10 (83.30) *p* < 0.001	23 (69.70) *p* < 0.001	558 (38.00)
Perforation of RV wall/ECHO finding	1 (4.50) *p* = 0.578	1 (8.30) *p* = 0.591	2 (6.10) *p* = 0.591	154 (10.50)

Abbreviations: LVEF—left ventricular ejection fraction, RA—right atrium, RV—right ventricle, RVSP—right ventricular systolic pressure, TVR—tricuspid valve regurgitation.

**Table 4:** TLE procedure complexity, efficacy, complications and mortality for any reason.

	Hemorrhagic Complication (Cardiac/Vascular Wall Tear)	Tricuspid Valve Damage	All Major Complications (Mixed Damages 1 Case)	Control Group (No Major Complications)
Groups of patients	A *N* = 22 Mean ± SD *n* (%)	B *N* = 12 Mean ± SD *n* (%)	C *N* = 33 Mean ± SD *n* (%)	D *N* = 1467 Mean ± SD *n* (%)
**TLE Procedure Complexity and Efficacy**				
Procedure duration (skin-to-skin)	104.4 ± 52.24 *p* < 0.001	81.33 ± 31.76 *p* = 0.004	94.61 ± 46.62 *p* < 0.001	60.93 ± 25.93
Procedure duration (sheath-to-sheath)	55.53 ± 55.93 *p* < 0.001	29.92 ± 21.16 *p* < 0.001	46.67 ± 48.69 *p* < 0.001	13.91 ± 21.32
Average time of single lead extr. (sheath-to-sheath/number of extracted leads)	25.62 ± 21.63 *p* < 0.001	16.69 ± 13.00 *p* < 0.001	21.79 ± 19.19 *p* < 0.001	8.40 ± 13.26
Technical problem during TLE (any)	14 (63.60) *p* < 0.001	8 (66.70) *p* < 0.001	21 (63.60) *p* < 0.001	321 (21.90)
Lead-to-lead binding (intraoperative diagnosis)	11 (50.00) *p* < 0.001	5 (41.70) *p* < 0.001	16 (48.50) *p* < 0.001	106 (7.20)
Block at venous entry site	4 (18.20 *p* = 0.497	3 (25.00) *p* = 0.497	7 (21.20) *p* = 0.134	165 (11.20)
Fracture of extracted lead	7 (31.80) *p* < 0.001	3 (25.00) *p* < 0.001	10 (30.30) *p* < 0.001	65 (4.40)
Byrd dilator torsion/collapse	2 (9.10) *p* = 0.544	4 (33.30) *p* = 0.544	6 (18.20) *p* = 0.544	61 (4.20)
Three or more technical problems	3 (13.60) *p* < 0.001	1 (8.30) *p* < 0.001	4 (12.10) *p* < 0.001	25 (1.70)
Use of Evolution (old and new) or TightRail	3 (13.60) *p* = 0.007	3 (25.00) *p* = 0.007	5 (15.20) *p* = 0.003	34 (2.30)
Use of lasso catheters/snares	5 (22.70) *p* < 0.001	3 (25.00) *p* < 0.001	8 (24.20) *p* < 0.001	47 (3.20)
Temporary pacing during procedure	3 (13.60) 0.348	5 (41.70) *p* = 0.348	8 (24.20) *p* = 0.3876	361 (24.60)
**TEE and Blood Pressure Monitoring**				
RAA pulling/drawing	15 (5) *p* < 0.001	3 (25.00) *p* < 0.001	18 (54.50) *p* = 0.012	472 (32.20)
TV pulling/drawing	6 (27.30) *p* < 0.001	11 (91.70) *p* < 0.001	16 (48.50) *p* < 0.001	100 (6.80)
RV wall pulling	10 (45.50) *p* = 0.015	8 (66.70) *p* = 0.015	17 (51.50) *p* < 0.001	317 (21.60)
Other lead pulling	10 (45.50)	5 (41.70) *p* < 0.001	15 (45.50) *p* < 0.001	116 (7.90)
Pulling/drawing of heart structures or other lead (possible multiple options)	1.864 ± 1.46 *p* < 0.001	2.250 ± 1.224 *p* < 0.001	2.00 ± 1.350 *p* < 0.001	0.670 ± 0.928
Max blood pressure drop during TLE [mm Hg]	54.43 ± 23.42 *p* < 0.001	38.89 ± 21.03 *p* < 0.001	48.38 ± 22.64 *p* < 0.001	20.79 ± 14.53
Significant blood pressure drop during TLE (different reasons)	13 (59.10) *p* < 0.001	3 (25.00) *p* < 0.001	15 (45.50) *p* < 0.001	137 (9.30)
**TLE Efficacy and Complications**				
Worsening TR for 1 degree	2 (9.10) *p* = 0.956	0 (0.00) *p* = 0.956	2 (6.10) *p* = 0.908	104 (7.10)
Worsening TR for 2 degrees	0 (0.00) *p* = 0.95	4 (33.30) *p* = 0.95	4 (12.10) *p* = 0.002	31 (2.10)
Worsening TR for 3 degrees	1 (4.50) *p* < 0.001	8 (66.70) *p* < 0.001	8 (24.20) *p* < 0.001	0 (0.00)
Tricuspid valve damage during TLE (severe)	0 (0.00) *N*	12 (100.0) *p* < 0.001	12 (36.40) *p* < 0.001	0 (0.00)
Procedure-related death (intra-, post-procedural)	0 (0.00) *N*	0 (0.00) *N*	0 (0.00) *N*	0 (0.00)
Clinical success	21 (95.50) *p* = 0.114	0 (0.00) *p* = 0.114	21 (63.60) *p* < 0.001	1463 (99.70)
Complete procedural success	20 (90.90) *p* = 0.322	0 (0.00) *p* = 0.322	20 (60.60) *p* < 0.001	1422 (96.90)
**Short-, Mid-and Long-Term Mortality after TLE (Any Reason)**				
First day (first 48 h)	0 (0.00) *p* = 0862	0 (0.00) *p* = 0.862	0 (0.00) *p* = 0.832	2 (0.14)
Mortality at 1 month after TLE (2–30 days)	0 (0.00) *p* = 0.78	0 (0.00) *p* = 0.780	0 (0.00) *p* = 0.993	23 (1.57)
Mortality at 1 year after TLE (31–365 days)	1 (4.55) 0.985	1 (8.33) 0.985	2 (6.06) 0.845	99 (6.75)
Mortality at 3 years TLE (366–1095 days)	1 (4.55) 0.855	0 (0.00) *p* = 0.855	1 (3.03) *p* = 0.841	116 (7.91)
Mortality > 3 years after TLE (> 1095 days)	0 (0.00) 0.673	1 (8.33) *p* = 0.673	1 (3.03) *p* = 0.888	60 (4.09)

Abbreviations: RAA—right atrial appendage, RV—right ventricle, TLE—transvenous lead extraction, TR—tricuspid regurgitation, TV—tricuspid valve.

## Data Availability

Readers can access the data supporting the conclusions of the study at www.usuwanieelektrod.pl.
